# Shear wave elastography for thyroid nodule evaluation in patients with chronic autoimmune thyroiditis

**DOI:** 10.1007/s12020-025-04159-1

**Published:** 2025-01-19

**Authors:** Monica Latia, Andreea Bena, Luciana Moisa-Luca, Ștefania Bunceanu, Dana Stoian

**Affiliations:** 1 Department of Doctoral Studies, Victor Babes University of Medicine and Pharmacy, Timisoara, Romania; 2 Center for Advanced Ultrasound Evaluation, Dr. D Medical Center, Timisoara, Romania; 3 Center of Molecular Research in Nephrology and Vascular Disease, Faculty of Medicine, Victor Babes University of Medicine and Pharmacy, Timisoara, Romania; 4 Discipline of Endocrinology, Second Department of Internal Medicine, Victor Babes University of Medicine and Pharmacy, Timisoara, Romania; 5 Endocrinology Unit, Pius Brinzeu Emergency Clinical Hospital, Timisoara, Romania

**Keywords:** Thyroid elastography, Thyroid nodule, Shear wave elastography, Chronic autoimmune thyroiditis, Thyroid cancer

## Abstract

**Purpose:**

Shear wave elastography (SWE) is a valuable tool in discerning the malignancy risk of thyroid nodules. This study investigates whether 2D-SWE can reliably differentiate malignant thyroid nodules in patients with chronic autoimmune thyroiditis (CAT), despite the challenges posed by fibrosis, which can increase tissue stiffness and complicate diagnosis.

**Methods:**

This retrospective observational study evaluated 130 thyroid nodules (91 benign, 39 malignant) in patients with underlying CAT using conventional ultrasound (B-mode) and 2D-SWE with SuperSonic Mach30 equipment (Supersonic Imagine, Aix-en-Provence, France). Pathology reports served as the reference standard.

**Results:**

Out of the 130 nodules, 30% were malignant, and 70% were benign. 2D-SWE proved to be an excellent distinguisher between benign and malignant nodules. Malignant nodules had significantly higher elasticity indices compared to benign nodules (mean elasticity index: 47.2 kPa vs. 18.1 kPa, *p* < 0.0001; maximum elasticity index: 75 kPa vs. 26.2 kPa, *p* < 0.0001). The mean elasticity index was the most reliable elastographic parameter (AUC 0.907, sensitivity 87.2% [95% CI: 77.3–94.0%], specificity 84.6% [95% CI: 75.4–91.5%], and NPV 93.9% for a cut-off value of 30.5 kPa).

**Conclusion:**

Our results confirm that 2D-SWE can accurately diagnose malignant thyroid nodules in cases with CAT (*p* < 0.0001), supporting its use as a complementary tool to conventional ultrasound.

## Introduction

Thyroid nodules are common, detected in up to 67% of individuals through ultrasonography [1]. Their prevalence increases with age and is associated with female sex, iodine deficiency, and head or neck radiation [2]. Thyroid cancer, the most common endocrine malignancy, is projected to be the fourth most common cancer by 2030 [1], with a rising incidence and malignancy rates of 7–15%, primarily in women [3]. Ultrasonography is the preferred imaging method for evaluating the neck region, due to the use of high-frequency, high-resolution transducers, being able to effectively characterize the morphology of thyroid gland as well as the presence and appearance of thyroid nodules [2, 4, 5]. Recently, ultrasound (US) elastography has emerged as a helpful additional tool that improves the diagnostic accuracy of traditional US evaluations. US elastography assesses tissue stiffness, which increases with pathological changes like inflammation or neoplastic growth. It helps distinguish benign from malignant thyroid nodules, with higher stiffness indicating malignancy [6, 7]. In 2D shear wave elastography (2D-SWE) the speed of these shear waves provides a quantitative assessment of tissue elasticity [8]. US elastography enhances thyroid nodule diagnosis and aids in better management decisions when combined with greyscale examination [7, 9–15].

Chronic autoimmune thyroiditis (CAT), or Hashimoto’s thyroiditis (HT), is an autoimmune thyroid disorder marked by chronic inflammation and anti-thyroid antibodies: anti-thyroid peroxidase (ATPO) and anti-thyroglobulin (ATG) antibodies. These antibodies damage thyroid tissue, leading to fibrosis and often hypothyroidism [16–18]. CAT primarily affects middle-aged women, with an incidence of up to 27% in adult women and 7% in men, increasing after age 50, and is linked to family histories of autoimmune diseases [17]. CAT features lymphoplasmacytic infiltration, germinal centers, and thyrocyte destruction, leading to inflammation, fibrosis, and altered thyroid elasticity. The fibrous variant, marked by extensive fibrosis, is the most recognized subtype [16–19].

US elastography holds significant potential in detecting CAT, due to its ability to identify the pathological features of the disease, particularly the presence of fibrosis leading to increased inelasticity [19, 20]. Several studies highlight the usefulness of quantitative SWE in diagnosing CAT both in the adult [20–24] and the pediatric population [25–29], as well as the possibility of using SWE to evaluate the degree of fibrosis associated with CAT. Cut off values for the diagnosis of CAT have been described and elastography could be useful in monitoring disease progression [15]. All current evidence shows that increased elasticity values are always found in patients with CAT compared to the normal thyroid, regardless of how advanced the disease is [19–23].

The current guidelines acknowledge US elastography as a valuable complementary method in the evaluation of thyroid nodules. However, in patients with autoimmune thyroid disease, the varying degrees of fibrosis associated with CAT are widely recognized as a significant limitation to the reliability of elastography in diagnosing thyroid nodules [8, 15, 30]. This study addresses this limitation by exploring the question: Does 2D-SWE effectively distinguish malignant thyroid nodules in patients with CAT, despite the challenges posed by fibrosis? By focusing on this specific subset of patients, this research not only reinforces the utility of SWE but also expands its applicability to a challenging clinical scenario where its use has been questioned.

## Materials and methods

### Patients

This retrospective observational study was conducted between January 2022 and June 2024 at a specialized endocrinology center in Timisoara, Romania (Dr. D Medical Center). It included 130 patients aged 18–84 years, all diagnosed with CAT and evaluated for thyroid nodules in the US department. The study was approved by the Ethics Committee of Victor Babeș University of Medicine and Pharmacy, Timișoara (approval no. 52/02.10.2023), and conducted in accordance with the ethical guidelines of the Helsinki Declaration. Written informed consent was obtained from all patients prior to their inclusion in the study.

### Inclusion and exclusion criteria

The study included patients diagnosed with CAT, confirmed through a combination of US findings and immunological assays showing elevated ATPO and/or ATG antibodies. Eligible participants had one or more thyroid nodules detected via conventional US and were further evaluated with US elastography. Only cases requiring surgical referral, based on current guidelines, were included. These criteria encompassed nodules classified as Bethesda categories IV to VI following fine needle aspiration (FNA), cases presenting with compressive symptoms, or multinodular goiters with high-risk US features. A complete histopathological report, considered the gold standard for diagnosis, was available for all included cases. For patients with multinodular goiter, only the nodule contributing to the pathological diagnosis was analyzed to ensure accuracy.

Patients were excluded if they did not have a confirmed diagnosis of CAT, if surgical referral was not warranted based on the established criteria, or if histopathological confirmation was unavailable. Additionally, nodules that did not meet the criteria for FNA or surgical intervention, or those that could not be reliably correlated with histopathological findings, were excluded.

### Conventional ultrasound and 2D shear wave elastography evaluation

The Aixplorer Mach 30 machine (Supersonic Imagine, Aix-en-Provence, France), equipped with an L 18-5 probe (linear, 5–18 MHz), was used for conventional B-mode thyroid US and 2D-SWE. The subject was positioned supine with a pillow under their neck to aid hyperextension. Minimal pressure was applied, and patients were instructed not to speak or swallow throughout the examination. Nodule characteristics (presence, location, size) were evaluated according to the European TIRADS (EU-TIRADS) [4, 5]. 2D-SWE was then performed during the same session, as it was found to be superior in evaluating nodules that coexist with autoimmune thyroid disease [31, 32]. The thyroid parenchyma’s elasticity was first assessed by placing the probe on one side of the neck and activating SWE mode. A color map was generated, ranging from blue (soft tissue) to red (hard tissue). The subject was instructed to hold their breath for 5 s, after which the image was frozen and elasticity measured using the Q-Box in kilopascals (kPa). The region of interest (ROI) was positioned approximately in the center of the thyroid lobe, preferably in hypoechoic areas if present. Six measurements were taken for each subject, with the elasticity value expressed in kPa [8, 15]. Next, for the evaluation of the thyroid nodule, the box was adjusted to encompass the entire lesion, with the probe held steady for 5 s to avoid external pressure. Images with compression artifacts, such as “finger-like” distortions or completely red images (indicating high stiffness) were excluded [33]. The ROI was positioned to cover the entire nodule [34], and five longitudinal measurements were recorded, with their mean used in the analysis of the maximum and mean elasticity index (EI), expressed in kPa. The QBox ratio was calculated by comparing two ROIs: one in the nodule (excluding artifacts or calcifications) and the other in the adjacent thyroid parenchyma or surrounding muscle, both at similar depths [8, 15, 35]. All the examinations were performed by the same sonographer, with over 10 years of expertise in US imaging.

### Cytology, surgical intervention, and pathology examination

Based on traditional US criteria for malignancy risk stratification, suspicious nodules meeting size thresholds were subjected to FNA [13]. Cytology results were reported according to the Bethesda system. Bethesda categories IV to VI were directed to surgery, as well as cases with compressive symptoms and multinodular goiters with high risk features on US. The pathological evaluation of surgical specimens categorized the histological subtypes according to the World Health Organization guidelines. The cytologist who reviewed all FNA samples was blinded to the US findings, and the pathologists who evaluated the cases were blinded to both the US and FNA results.

### Statistical analysis

Data were analyzed using Microsoft Excel and MedCalc Statistical Software V19.4 (MedCalc Software Ltd., Flanders, Belgium). The distribution of variables was tested for normality using the Shapiro–Wilks test. As most of the data were not normally distributed, continuous variables between the benign and malignant groups were compared using the Mann–Whitney U test, and results are presented as median with interquartile range (IQR). Categorical variables were analyzed using the Chi-square test. A significance level of *p* < 0.05 was applied for all comparisons. To evaluate the diagnostic accuracy of the elastographic parameters, receiver operating characteristic (ROC) curve analysis was performed. The area under the ROC curve (AUC), sensitivity, specificity, positive predictive value (PPV), and negative predictive value (NPV) were calculated for each parameter, and the optimal cut-off points were determined using Youden’s index. Correlations between histopathological results and other parameters were assessed using Spearman’s rank correlation. Correlation coefficients (r) were reported, with p-values indicating the strength of associations. Significant correlations were highlighted, with coefficients greater than 0.4 suggesting a moderate relationship and those above 0.6 indicating a strong relationship.

## Results

The characteristics of the study group, comparing benign and malignant thyroid nodules subgroups are displayed in Table 1. The table includes demographic data, side involvement, thyroid function, and autoimmunity parameters (TSH, ATPO antibodies, ATG antibodies). No statistically significant differences were found between the benign and malignant groups for most parameters, except for the number of nodules.

**Table 1:** Characteristics of the study group

	All nodules Median (IQR)	Benign	Malignant	*p* value
Number of nodules	130	91	39	**<0.0001**
Age	58 (50–68)	59 (51–68)	57.5 (38–67)	0.198
Gender (F/M)	110/20	78/13	32/7	0.563
Affected lobe (Right/left)	72/58	43/48	18/21	0.930
TSH (μIU/mL)	3.160 (2.210–4.060)	3.2 (1.9–4)	2.8 (2.4–3.7)	0.7934
ATPO Ab (IU/mL)	105.6 (78.5–1300)	98.7 (65–1230)	110.4 (73.2–1300)	0.673
ATG Ab (IU/mL)	163 (10.7–1530)	(11.1–1530)	157 (9–1530)	0.830

*TSH* thyroid stimulating hormone, *ATPO Ab* anti-thyroid peroxidase antibodies, *ATG Ab* anti-thyroid globulin antibodies, *IQR* interquartile range; *p* significance level (Mann-Whitney)

*P* values that are statistically significant are shown in bold

Table 2 depicts the US-based characteristics of the thyroid nodules and its surrounding parenchyma. Significant differences were found in the conventional US risk-assessment (TIRADS scores), mean and maximum nodule elasticity indices (EIs), and the nodule-to-thyroid shear wave ratio (*p* < 0.0001). We did not detect significant differences between EIs of thyroid parenchyma in benign versus malignant cases.

**Table 2:** Conventional ultrasound risk-assessment and elastography characteristics of thyroid nodules

	All nodules	Benign	Malignant	*p* value
Thyroid volume (ml)	19 (12–28)	19 (12–29)	17 (12–28)	0.4128
TIRADS	4a (4a–4b)	4a (4a–4b)	4b (4b–4c)	**<0.0001**
Mean EI RTL (kPa)	18.9 (13.8–23.9)	18.5 (13.5–23.9)	19.6 (15.1–24.5)	0.3293
Mean EI LTL (kPa)	22.1 (15.3–23.6)	18.1 (14.8–23.3)	19.9 (17.3–24.6)	0.0754
Mean thyroid EI (kPa)	19.6 (15.1–24.1)	19.4 (14.3–23.7)	19.9 (16–24.9)	0.2086
Mean Nodule EI (kPa)	30.29 (15–36.9)	18.1 (12.6–24.5)	47.2 (34–63.2)	**<0.0001**
Max EI RTL (kPa)	23.8 (18.3–30.7)	23.7 (18.1–30)	26 (20.2–31.9)	0.1434
Max EI LTL (kPa)	24.9 (20.2–30.7)	24.4 (19.5–28.7)	27.8 (21.8–31.4)	0.0588
Max thyroid EI (kPa)	24.7 (20.5–31.2)	24.5 (19.2–29.7)	26.5 (21.8–32)	0.0827
Max Nodule EI (kPa)	37 (23.3–57.4)	26.2 (21.9–41.3)	75 (48.1–89.8)	**<0.0001**
N/T Mean EI ratio	1.22 (0.8–2)	0.95 (0.7–1.5)	2.4 (1.6–3.2)	**<0.0001**
Depth (cm)	1.5 (1.3–1.8)	1.5 (1.3–1.8)	1.6 (1.2–1.87)	0.9634

*TIRADS* thyroid imaging reporting and data system, *N/T ratio* nodule/thyroid parenchyma shear wave ratio, *RTL* right thyroid lobe, *LTL* left thyroid lobe, *EI* elasticity index

*P* values that are statistically significant are shown in bold

**Fig. 1:**
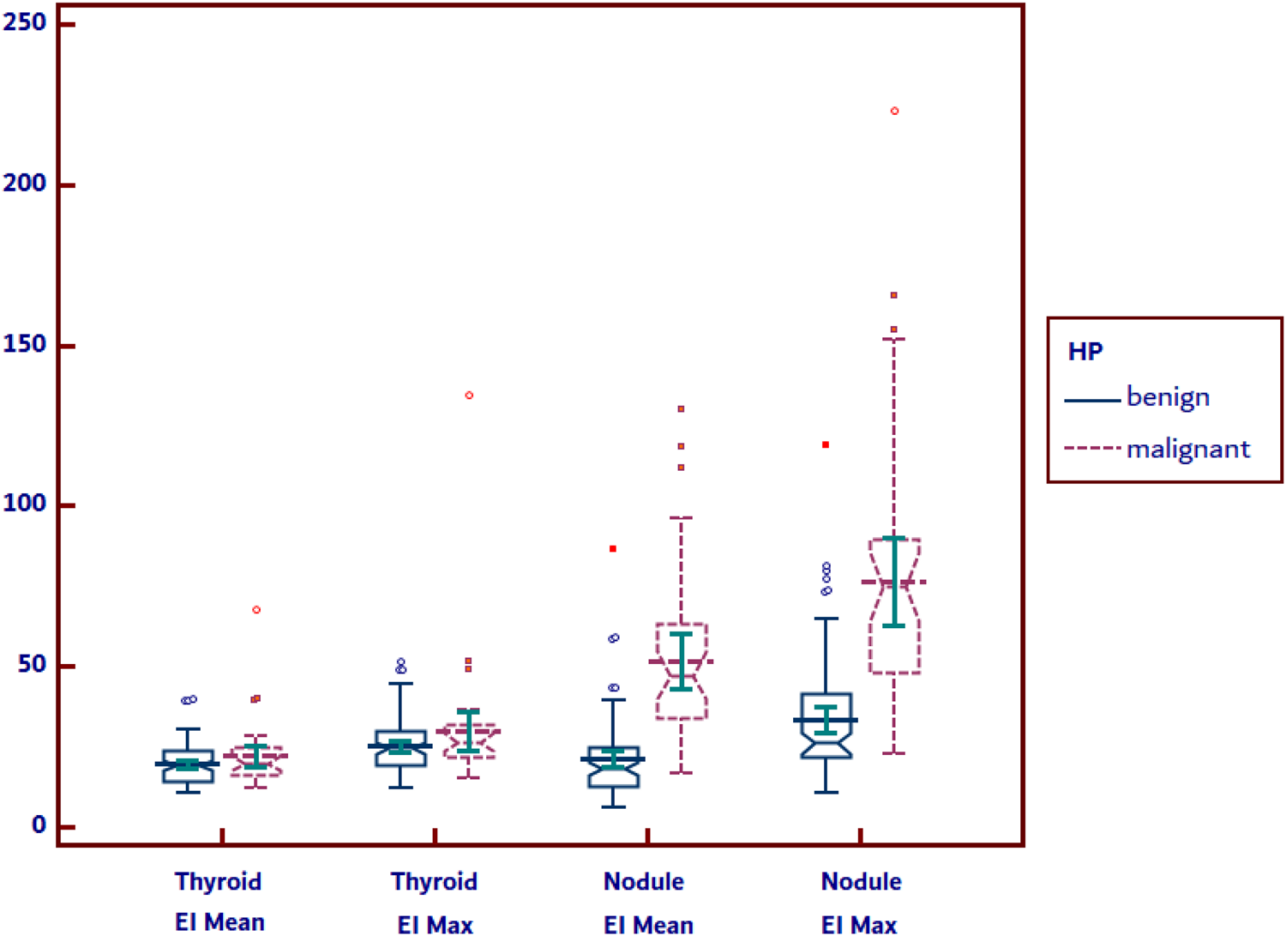
Graphical display of elasticity indices (mean and max) of thyroid nodules and thyroid parenchyma in cases with benign and malignant pathology results

The EIs (mean and maximum) for both thyroid nodules and thyroid parenchyma in cases with benign and malignant histopathological results are illustrated in Fig. 1. In the case of thyroid nodules, the EIs are significantly higher in malignant nodules compared to benign ones for both maximum and mean EI (*p* < 0.0001).

**Fig. 2:**
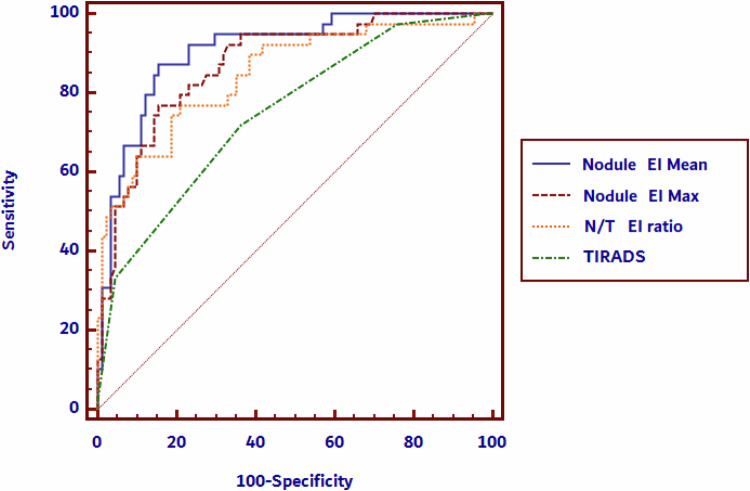
Graphical display of the comparison of AUCs for nodule elastography parameters and TIRADS

Figure 2 illustrates a comparison of the area under the curve (AUC) values for various elastographic parameters in differentiating benign from malignant thyroid nodules. The parameters compared include the mean and maximum EIs of the nodule, the ratio of nodule to thyroid parenchyma stiffness (N/T EI ratio) and the TIRADS classification. The AUCs for these parameters are displayed in Table 3. All elastography parameters showed higher diagnostic performance compared to B-mode risk assessment (TIRADS).

**Table 3:** AUC parameters for nodule elastography parameters and TIRADS

	AUC	Sensitivity (%)	Specificity (%)	NPV (%)	PPV (%)	Cut-off value
Mean Nodule EI	0.907	87.2	84.6	93.9	70.8	>30.5 kPa
Max Nodule EI	0.873	76.9	84.6	89.5	68.2	>47.4 kPa
N/T ratio	0.847	76.9	79.1	88.9	61.2	>1.6
TIRADS	0.749	71.8	63.7	84.1	46	>4

*AUC* area under the curve, *TIRADS* thyroid imaging reporting and data system, *N/T ratio* nodule/thyroid parenchyma shear wave ratio, *EI* elasticity index, *NPV* negative predictive value, *PPV* positive predictive value

The statistical analysis shows that the mean elasticity index of the nodule (Mean nodule EI) outperforms both the maximum elasticity index (Max nodule EI) and the ratio of nodule to thyroid stiffness (N/T EI ratio), with significant differences between the AUCs (*p* = 0.0360 and *p* = 0.0130, respectively). Additionally, the Mean nodule EI has a significantly higher diagnostic performance compared to TIRADS (*p* = 0.0025). However, the differences between the diagnostic performance of the Max nodule EI and N/T EI ratio, as well as N/T EI ratio and TIRADS, were not statistically significant (*p* = 0.3367 and *p* = 0.1178, respectively); thus the Mean nodule EI is the most reliable elastographic parameter in our analysis for distinguishing malignant from benign nodules.

Importantly, Mean nodule EI with a cut-off value for malignancy of 30.5 kPa detects the most cancers because it has the highest sensitivity (87.2%) and negative predictive value (NPV) (93.9%), meaning it correctly identifies a larger proportion of malignant cases and has fewer false negatives compared to TIRADS, which has lower sensitivity (71.8%) and NPV (84.1%).

Among the 39 differentiated carcinomas, 35 were papillary and 4 were follicular. The Mean nodule EI for follicular carcinoma was 37.6 ± 11.9 kPa, while for papillary carcinoma it was 53.1 ± 26.9 kPa, with no statistical significance (*p* = 0.26). However, it is notable that all 35 papillary carcinomas had a mean elasticity index above our cut-off for malignancy of 30.5 kPa.

The correlation (Spearman correlation coefficient r) between histopathological outcomes and elastographic parameters, including maximum and mean EIs in both normal and pathological thyroid tissue, is displayed in Table 4. The significant correlations are highlighted in bold, demonstrating a strong association between increased tissue stiffness and malignant nodules. The elasticity index of thyroid parenchyma did not correlate with pathology results.

**Table 4:** Correlation table

		HP	EI max N	EI mean N	TIRADS	EI N/T ratio	EI max T	EI mean T	TSH	Age
EI max N	r P	**0.592** <**0.0001**								
EI mean N	r P	**0.646** <**0.0001**	**0.901** <**0.0001**							
TIRADS	r p	**0.414** <**0.0001**	**0.401** <**0.0001**	**0.391** <**0.0001**						
EI N/T ratio	r p	**0.552** <**0.0001**	**0.809** <**0.0001**	**0.885** <**0.0001**	**0.257** **0.003**					
EI max T	r p	0.153 0.082	**0.207** **0.018**	**0.206** **0.018**	**0.285** **0.001**	−**0.211** **0.015**				
EI mean T	r p	0.111 0.209	0.114 0.195	0.137 0.119	**0.226** **0.009**	−**0.308** **0.0004**	**0.932** <**0.0001**			
TSH	r p	−0.032 0.795	−0.224 0.070	−0.237 0.055	0.081 0.519	−0.245 0.047	−0.052 0.677	−0.034 0.786		
Age	r p	−0.160 0.200	−0.149 0.233	−0.219 0.077	−0.028 0.823	−0.090 0.473	−0.142 0.255	−0.158 0.204	−0.129 0.300	
TV	r p	−0.072 0.414	−0.003 0.970	0.001 0.995	−0.070 0.430	−0.084 0.344	0.075 0.398	0.068 0.442	0.122 0.330	−0.204 0.099

*HP* pathology result, *TIRADS* thyroid imaging reporting and data system, *N/T ratio* nodule/thyroid parenchyma shear wave ratio, *EI* elasticity index, *N* nodule, *T* thyroid, *TSH* thyroid stimulating hormone, *r* Spearman rank correlation coefficient

*P* values that are statistically significant are shown in bold

## Discussion

CAT involves chronic thyroid inflammation, characterized by lymphocytic infiltration and varying degrees of fibrosis that lead to structural changes such as firm consistency, an inhomogeneous thyroid appearance, and nodule development [19, 20]. While conventional US is commonly used to evaluate CAT, it primarily suggests the condition and requires laboratory tests for definitive diagnosis. Elastography complements classical US by providing detailed assessments of fibrotic tissue and thyroid nodules, with SWE demonstrating high sensitivity (85.2%) and specificity (94.2%) for predicting malignant nodules [36, 37]. By highlighting significant elasticity index differences (*p* < 0.001) between benign and malignant nodules, SWE enhances diagnostic accuracy and aids in reducing unnecessary biopsies, improving management outcomes [15, 38].

In CAT patients, SWE-measured elasticity values are influenced by fibrosis and inflammation. Studies consistently report elevated stiffness in CAT-affected thyroid tissues, typically ranging from 18 to 35 kPa, compared to normal thyroid tissue values below 17 kPa [21, 22, 25, 36]. In our study, thyroid parenchyma elasticity values (mean EI: 19.6 kPa, max EI: 24.7 kPa) aligned with literature. However, our findings revealed no significant differences in thyroid parenchyma elasticity between malignant and benign cases, indicating that background parenchymal elasticity does not interfere with nodule categorization.

The application of elastography in patients with CAT presents unique challenges due to the increased stiffness of the thyroid tissue. Both fibrosis and malignancy can elevate stiffness values, complicating differentiation between benign and malignant nodules. This overlap in stiffness measurements is evident in the variability of cut-off values for elasticity indices in CAT populations, reflecting the diagnostic complexity. While many studies exclude CAT patients due to these limitations, leaving a critical gap in the literature, SWE remains a highly effective tool for evaluating thyroid nodules in this group, with elasticity values exceeding 35 kPa suggestive of malignancy. SWE sensitivity (85.2%) and specificity (94.2%) persist even in fibrotic thyroid parenchyma, with significant elasticity differences (*p* < 0.001) between benign and malignant nodules [31, 32, 34].

The findings in this study align with prior research, showing significantly higher EIs (mean and maximum) in malignant nodules compared to benign ones (*p* < 0.0001) [31, 32]. All elastography parameters (mean and maximum EIs and N/T EI ratio) outperformed B-mode risk assessment (EU-TIRADS), highlighting the efficacy of 2D-SWE in thyroid nodule evaluation [39]. The sensitivity and NPV of EU-TIRADS in this study were lower than reported in previous studies, such as Grani et al. [40]. This difference may be explained by the high prevalence of autoimmune thyroid disease in our cohort, as CAT can alter US features and impact the performance of TIRADS classifications.

The 2D-SWE evaluation identified mean elasticity index (Mean nodule EI) as the most reliable parameter for distinguishing malignant from benign nodules, surpassing maximum elasticity index (Max nodule EI) and nodule-to-thyroid stiffness ratio (N/T EI ratio). While there is no consensus favoring either Mean EI or Max EI, WFUMB guidelines recommend using a cut-off of over 40 kPa for malignancy, with benign nodules typically below 30–40 kPa [15, 39, 41]. Studies using similar techniques report Mean EI cut-offs ranging from 35 kPa to as high as 49 kPa, achieving sensitivity of 78% and specificity of 81% [42–44]. Lower cut-offs, such as 23 kPa, have been noted in studies that included entire nodules within the ROI [45]. In CAT patients, some studies suggest adjusting cut-offs to account for fibrotic and inflammatory tissue, though 40 kPa remains a widely accepted benchmark [15, 34].

For this study, a cut-off of 30.5 kPa for Mean nodule EI achieved a sensitivity of 87.2%, specificity of 84.6%, and a NPV of 93.9%, outperforming EU-TIRADS, which showed a sensitivity of 71.8%, specificity of 84.6%, and NPV of 84.1%. While the Mean nodule EI demonstrated strong diagnostic performance individually, we recommend including this elastographic parameter into the TIRADS score. As described in multiple previous studies, nodules with increased stiffness and EIs exceeding the malignancy cut-off value should be upgraded to a higher TIRADS risk category. This integration refines the diagnostic accuracy of standalone TIRADS models, enhancing risk stratification [46, 47]. Therefore, we recommend using 2D-SWE in future evaluations of this specific patient subgroup, specifically to upgrade nodules to a higher TIRADS risk class when the mean EI exceeds the 30.5 kPa cut-off value. Such an approach holds promise for improving the precision of malignancy risk assessment and optimizing clinical decision-making.

A significant advantage of SWE is its ability to provide standardized, quantitative values, making it more reproducible and less operator-dependent than other US methods. However, SWE is still susceptible to artifacts, particularly from external compression, which can affect measurements in challenging areas like the thyroid isthmus. Ensuring accuracy requires trained, experienced operators to minimize errors in nodule evaluation. Although currently considered an ancillary technique in European guidelines due to its limited availability and accessibility, numerous studies highlight its diagnostic accuracy and value in thyroid nodule evaluation, especially in challenging cases. These findings support its potential as a complementary tool to improve diagnostic precision.

We identified several limitations in our study. All patients included in the study had normal TSH values (euthyroid or euthyroid under supplemental treatment), preventing the assessment of elasticity variations related to hypothyroidism. Additionally, the lack of a control group narrowed the focus to evaluating the diagnostic accuracy of 2D-SWE in thyroid nodules coexisting with CAT. The retrospective design introduced potential selection bias, and the relatively small sample size reduced the statistical power and generalizability of the results. The uneven distribution of subgroups, particularly the small size of the compressive symptoms group, further limited statistical comparisons. Conducting the study in a single specialized endocrinology center also impacted the broader applicability of the findings. Future prospective, multicenter studies with larger cohorts are needed to validate these results and extend their relevance across diverse patient populations.

In this study, the pathology report was used as the gold standard to ensure diagnostic accuracy, given its definitive nature. However, this approach may have increased the malignancy rate due to the selection of surgically treated nodules. The study population, focused on thyroid nodules with coexistent CAT, represents a specific subgroup, which may limit generalizability. This targeted approach aimed to address diagnostic challenges within this subset. Further research in more heterogeneous populations is necessary to confirm the findings.

Advanced imaging technologies like 2D-SWE may not be widely accessible in resource-limited environments, where conventional US remains a primary diagnostic tool, with 2D-SWE serving as a complementary technique when available. To enhance accessibility, the development of cost-effective elastography solutions or alternative diagnostic strategies tailored to resource-limited settings is essential. Further research is needed to explore scalable approaches that integrate elastography’s benefits while addressing financial and logistical constraints.

Given its demonstrated utility in accurately differentiating thyroid nodules, 2D-SWE is particularly beneficial for nodules with intermediate and high-risk US features. In resource-limited settings, a tiered approach could be effective, starting with initial screenings using basic US technology at primary care centers, followed by referrals to specialized centers with elastography capabilities. This strategy would ensure that patients in low-resource environments can still benefit from 2D-SWE, enhancing diagnostic confidence and improving outcomes.

## Conclusions

This study evaluated the diagnostic accuracy of 2D-SWE in assessing thyroid nodules coexisting with CAT. The findings demonstrate that 2D-SWE enhances diagnostic confidence when used alongside conventional US. The Mean nodule EI value emerged as the most reliable parameter, predicting malignancy with a sensitivity of 87.2%, specificity of 84.6%, and a NPV of 93.9%. While these results support the utility of 2D-SWE in identifying malignant nodules in patients with CAT, further studies are warranted to validate these findings in larger cohorts and diverse clinical settings.

## Data Availability

No datasets were generated or analysed during the current study.
